# Insulin exposure mitigates the increase of arterial stiffness in patients with type 2 diabetes and albuminuria: an exploratory analysis

**DOI:** 10.1007/s00592-019-01351-4

**Published:** 2019-05-22

**Authors:** Daniel Gordin, Markku Saraheimo, Jaana Tuomikangas, Aino Soro-Paavonen, Carol Forsblom, Karri Paavonen, Birgit Steckel-Hamann, Valma Harjutsalo, Loizos Nicolaou, Imre Pavo, Veikko Koivisto, Per-Henrik Groop

**Affiliations:** 1 Folkhälsan Institute of Genetics, Folkhälsan Research Center, Biomedicum Helsinki (C318b), Haartmaninkatu 8, 00290 Helsinki, Finland; 2 Abdominal Center Nephrology, University of Helsinki and Helsinki University Hospital, Helsinki, Finland; 3 Research Programs Unit, Diabetes and Obesity, University of Helsinki, Helsinki, Finland; 4 Department of Medicine, University of Helsinki and Helsinki University Hospital, Helsinki, Finland; 5 Eli Lilly & Co Export SA, Geneva, Switzerland; 6 Chronic Disease Prevention Unit, National Institute for Health and Welfare, Helsinki, Finland; 7 ClinBAY, Genappe, Belgium; 8 Eli Lilly & Co, Vienna, Austria; 9 Eli Lilly & Co, Helsinki, Finland; 10 Department of Diabetes, Central Clinical School, Monash University, Melbourne, VIC Australia

**Keywords:** Type 2 Diabetes, Pulse wave velocity, Arterial stiffness, Insulin resistance, Albuminuria, Diabetic nephropathy, Diabetic kidney disease

## Abstract

**Aims:**

Insulin possesses both vasodilatory and sympathomimetic activities. The aim was to examine the relationship between changes in insulin exposure and arterial stiffness in type 2 diabetes (T2D).

**Methods:**

Patients with T2D with (*n* = 22) or without (*n *= 24) albuminuria, and non-diabetic controls (*n* = 25) were randomized to a crossover study having a breakfast with or without pre-meal rapid-acting insulin. Pulse wave velocity (PWV) was measured at 30 min before and at 60-min intervals up to 240 min after the breakfast.

**Results:**

At baseline, both postprandial aortic (*p *= 0.022) and brachial (*p *= 0.011) PWV were higher in individuals with T2D than in healthy controls irrespective of the presence of albuminuria. In patients with albuminuria, weight-adjusted insulin dose correlated inversely with the excursion of the aortic (*r *= − 0.412, *p *= 0.006) and brachial (*r* = − 0.372; *p *= 0.014) PWV. Similarly, circulating endogenous insulin concentrations correlated inversely with the aortic (*r *= − 0.347, *p *= 0.026) and brachial (*r *= − 0.622, *p *= <0.001) PWV. No correlations between insulin and PWV were observed in patients without albuminuria or in healthy controls.

**Conclusions:**

The inverse correlation between insulin and PWV in T2D with albuminuria may reflect a vasorelaxing effect of insulin.

**Clinical trial registration number:**

The study was registered (clinicaltrials.gov) with the identifier of NCT01159938.

## Introduction

The role of insulin in enhancing peripheral blood flow as well as increasing arterial diameter in large arteries is well established [1–3]. The vasodilatory effect of insulin can be blocked by inhibiting nitric oxide synthesis highlighting the endothelial effects of insulin [4]. It is not known if the vasodilatory effect of insulin is preserved in patients with diabetic complications. An open but important question is whether albuminuria, which is often related to autonomous neuropathy and endothelial dysfunction, influences the effect of insulin on blood flow and/or arterial stiffness [5–8]. In the absence of autonomic neuropathy, there is synchrony between vasodilatory and vasoconstrictive effects of insulin in maintaining appropriate blood flow. We have previously shown that postprandial hyperglycemia increases brachial pulse wave velocity (PWV) in albuminuric but not in normoalbuminuric patients with type 2 diabetes (T2D) [9]. Now we analyzed whether there is any association between postprandial hyperinsulinemia and the stiffness of large (aortic) and middle-sized (brachial) arteries in individuals with T2D, and if so, are there differences between T2D patients with or without albuminuria.

## Subjects, study design and methods

### Subjects

Twenty-two patients with type 2 diabetes with and 24 patients without albuminuria, and 25 non-diabetic control subjects were studied after a high-carbohydrate meal. Eligible individuals were male between 45 and 70 years of age, had not smoked in the 12 h before the visits, and had not experienced cardiovascular event such as coronary heart disease, stroke or peripheral vascular disease. All 24 patients with albuminuria, 21/22 patients without albuminuria and 5/20 control subjects were on antihypertensive medication. ACE inhibitors/ATII blockers were used by 5/10 patients, with albuminuria, 2/5 patients without albuminuria, and 6/1 control subject. Cholesterol-lowering agents (mostly statins) were used by 73% of patients with albuminuria, 92% of patients without albuminuria and 8% of the control subjects as described previously [9]. Their clinical characteristics are shown in Table 1. The study protocol is in accordance with the Declaration of Helsinki as revised in 2000, and approved by the local ethics committee. Written informed consent was obtained from each patient.

**Table 1:** Subject characteristics

	Non-diabetic controls	Patients with albuminuria	Patients with normal UAER
	*N *= 25	*N *= 22	*N *= 24
Age (years)	59 ± 7	61 ± 5 (0.148)	64 ± 5 (0.004)
BMI (kg/m^2^)	27 ± 3	34 ± 5 (< 0.001)	32 ± 6 (< 0.001)
Disease duration (years)	NA^a^	12 ± 4	16 ± 8
UAER (ug/min)	NA^a^	273 ± 476	5 ± 5
HbA_1c_ (%) (mmol/mol)	5.4 ± 0.3	7.8 ± 1.3 (< 0.001)	7.4 ± 1.1 (< 0.001)
	35 ± 1.5	62 ± 6.5	57 ± 5.5
Total cholesterol (mmol/l)	5.3 ± 0.8	4.1 ± 0.7 (< 0.001)	4.3 ± 0.9 (< 0.001)
Triglycerides (mmol/l)	1.3 ± 0.5	1.5 ± 0.8 (0.554)	1.6 ± 1.5 (0.401)
Insulin dose (U)			
Lispro	NA	13 ± 14	11 ± 6
Basal insulin. high PP glucose	NA	80 ± 53	61 ± 49
Basal insulin. low PP glucose	NA	78 ± 53	61 ± 50

Values inside the brackets illustrate *p* values for pairwise comparison to healthy volunteers

*BMI* body mass index, *UAER* urinary albumin excretion

^a^Not analyzed for statistical difference

### Study design

The study was performed over two single-day clinical visits. Patients (stratified by the presence or absence of albuminuria) were randomized to two treatment schedules: either insulin lispro was or was not injected subcutaneously before a standard breakfast. The patients injected their basal insulin (insulin glargine or detemir) as usual before the study. Healthy individuals were studied only once. Blood samples were drawn and PWV was measured 30 min before and 60, 120, 180, and 240 min after the breakfast meal. The design and the composition of the meals have already been published in the previous paper [9]. In brief, this study was done in three single-day visits. During visit 1, the patients with diabetes were randomized blindly (albuminuria vs normal UAER) to have insulin lispro subcutaneously before breakfast meal either during visit 2 or visit 3. The breakfast included 500 kcal, 60% carbohydrate, 20% protein and 20% fat. The day insulin lispro was injected, the rise in glucose was less (low-glucose study) than on the day lispro was not injected (high-glucose study). Insulin dosing was based on the patient’s normal morning insulin dose and the energy content of the meal. If the study breakfast was larger than the patient’s normal breakfast, insulin lispro dose was increased by 20–50% at the discretion of the investigator.

### Methods

To measure the arterial stiffness in the large (carotid–femoral or aortic) and intermediate-sized (carotid–radial or brachial) arteries, PWV pressure waveforms were recorded sequentially at the carotid, femoral, and radial arteries with a high-fidelity micromanometer (SPC-301; Millar Instruments: SphygmoCor, Sydney Australia). With a simultaneous EKG recording of the R-wave as reference frame, the software calculated the PWV [10].

Blood glucose was measured by the glucose oxidase method using a HemoCue 201 m (Hemocue, Ängelholm, Sweden). Insulin was quantitated with a immunoelectrochemiluminometric assay on the Modular Analytics E170 analyzer (Roche Diagnostics, Mannheim, Germany). The detection limit of the assay is 0.2 mU/L. Intra-assay coefficient of variation is < 2% and inter-assay variation < 5% at 7–400 mU/L. Cross-reaction with insulin analogues lispro, aspart, glargine, detemir and glulisine is < 0.1% [11, 12]. The assay is calibrated against WHO 1st IRP NIBSC 66/304.

### Statistical analyses

Pearson correlation coefficient was used to summarize the association between insulin and PWV. The observed area under the curve (AUC) (AUC_0–4h_) not adjusted for baseline values as in our previous paper [9]) was calculated for PWV and the insulin concentrations. The least square (LS) means, obtained from an ANCOVA model (including age and BMI as covariates and the subject group as factor) were summarized separately by subject group and glycemic condition (i.e., low or high). The LS means were compared between the subject groups and the significance was assessed at the two-sided 5% level. Finally, to test whether insulin has a similar effect on PWV (similar slopes) in patients with and without albuminuria and in the control group, the interaction term between the groups and insulin as continuous measurement was incorporated in the model. While the individuals with T2D were studied during two periods (i.e., under high- and low-postprandial glucose conditions), the non-diabetic controls were studied only for one period. The model predicted mean data per time point for the control group were different between the two glycemic conditions due to the effect of adjusting for covariates. Statistical analyses were performed using SAS 9.4 version (SAS Institute Inc, Cary, NC, USA).

## Results

### Blood glucose

In the two diabetes patient groups combined, the LS mean ± SE glucose level was 6.8 ± 0.2 mmol/L before breakfast. The peak postprandial rise in blood glucose after breakfast was lower with (3.3 ± 0.3 mmol/L, *p *= 0.001) than without (5.3 ± 0.3 mmol/L) pre-meal lispro injection (*p *= 0.001). While the glucose levels were significantly higher throughout the study in both diabetes groups as compared to healthy controls (*p *< 0.01–0.001), there were no differences in postprandial glucose levels between patients with or without albuminuria [9].

### Insulin dosages and concentrations

There was no significant difference in the mean doses (U ± SD) of insulin lispro and basal insulin (glargine or levemir) in patients with albuminuria (lispro 13.4 ± 13.7 + basal 80.1 ± 53.3 vs 77.7 ± 53.4 basal only in low- and high-glucose studies, respectively) compared to patients without albuminuria (lispro 11.2 ± 6.1 + basal 60.8 ± 49.5 vs 61.3 ± 50.0 basal only). Neither was there any significant difference in the LS mean AUC (± SE) of serum insulin concentrations in the high- and low-glucose studies, respectively, in patients with (96.4 ± 18.6 and 84.1 ± 18.3 mU * h/L) or without albuminuria (119.2 ± 17.2 and 102.1 ± 17.4 mU * h/L), or between the diabetes patient groups. In healthy individuals, the mean serum insulin AUC was 109.7 ± 19.7 mU * h/L, not significantly different from the two diabetes groups. There was no significant difference between the diabetic groups in insulin AUC as such, or when AUC was adjusted by weight.

### PWV

At baseline, the LS mean (± SE) of AUC PWV in the aorta was higher in patients with (42.3 ± 2.4 and 41.9 ± 2.27 m * h/s) and without albuminuria (41.2 ± 2.3 and 41.6 ± 2.3 m * h/s) as compared to healthy controls (34.3 ± 2.3 and 36.0 ± 2.3 m *h/s, *p *= 0.022 and 0.072), while there was no difference between the two diabetes subgroups. Similarly, the LS mean (± SE) of AUC brachial PWV was higher in patients with (32.1 ± 1.1 and 32.9 ± 1.2 m * h/s) and without albuminuria (30.6 ± 1.0 and 31.2 ± 1.2 m * h/s) as compared to healthy controls (27.7 ± 1.1 and 27.6 ± 1.3 m * h/s, *p *= 0.011 and 0.009), while there was no difference between the two diabetes subgroups.

### Correlation between insulin and PWV

**Fig. 1:**
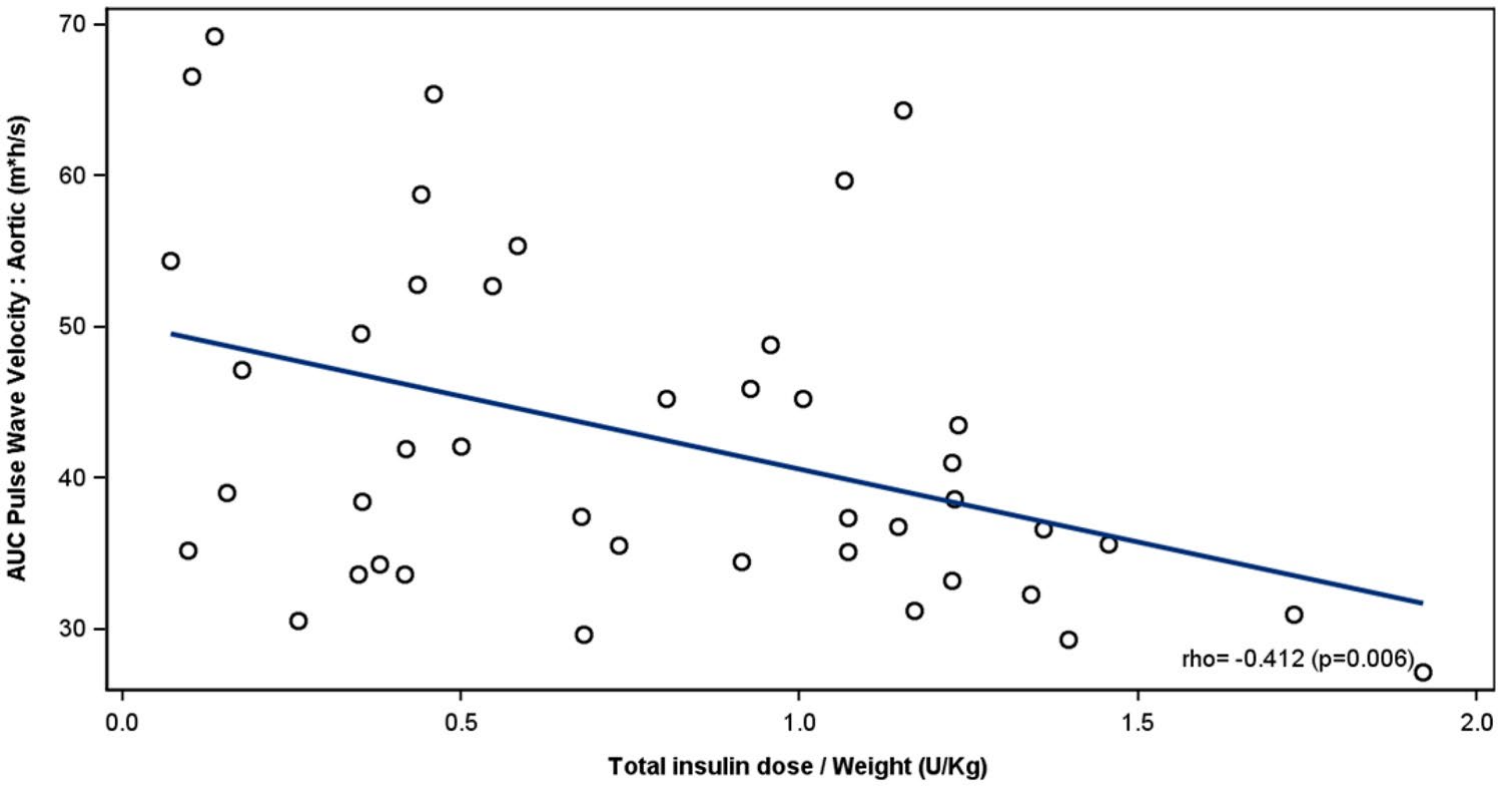
Correlation between AUC aortic PWV and total insulin dose/weight in patients with type 2 diabetes with albuminuria. For each patient, a study with and without pre-meal lispro is included

**Fig. 2:**
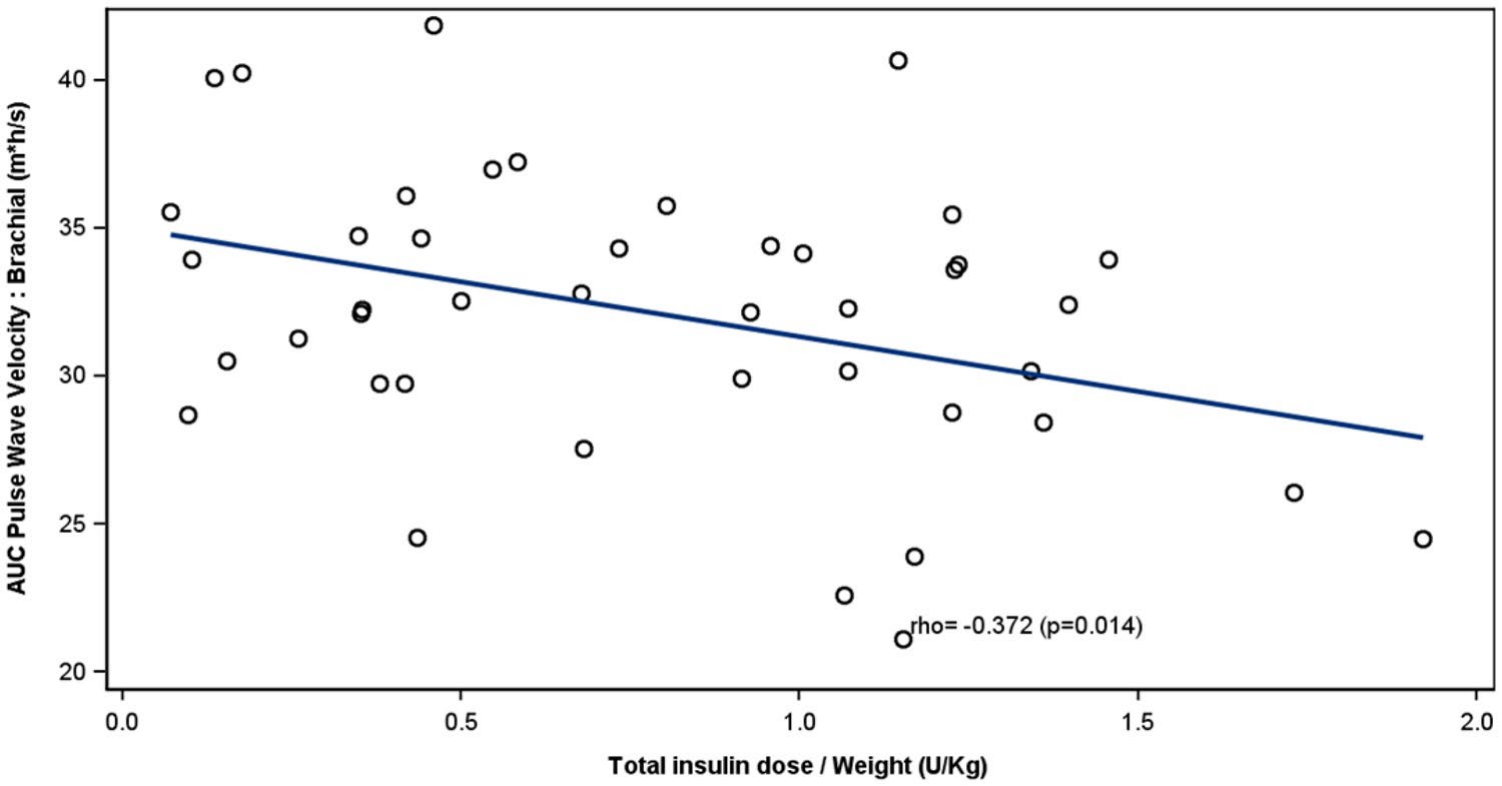
Correlation between AUC brachial PWV and total insulin dose/weight in patients with type 2 diabetes with albuminuria. For each patient, a study with and without pre-meal lispro is included

**Fig. 3:**
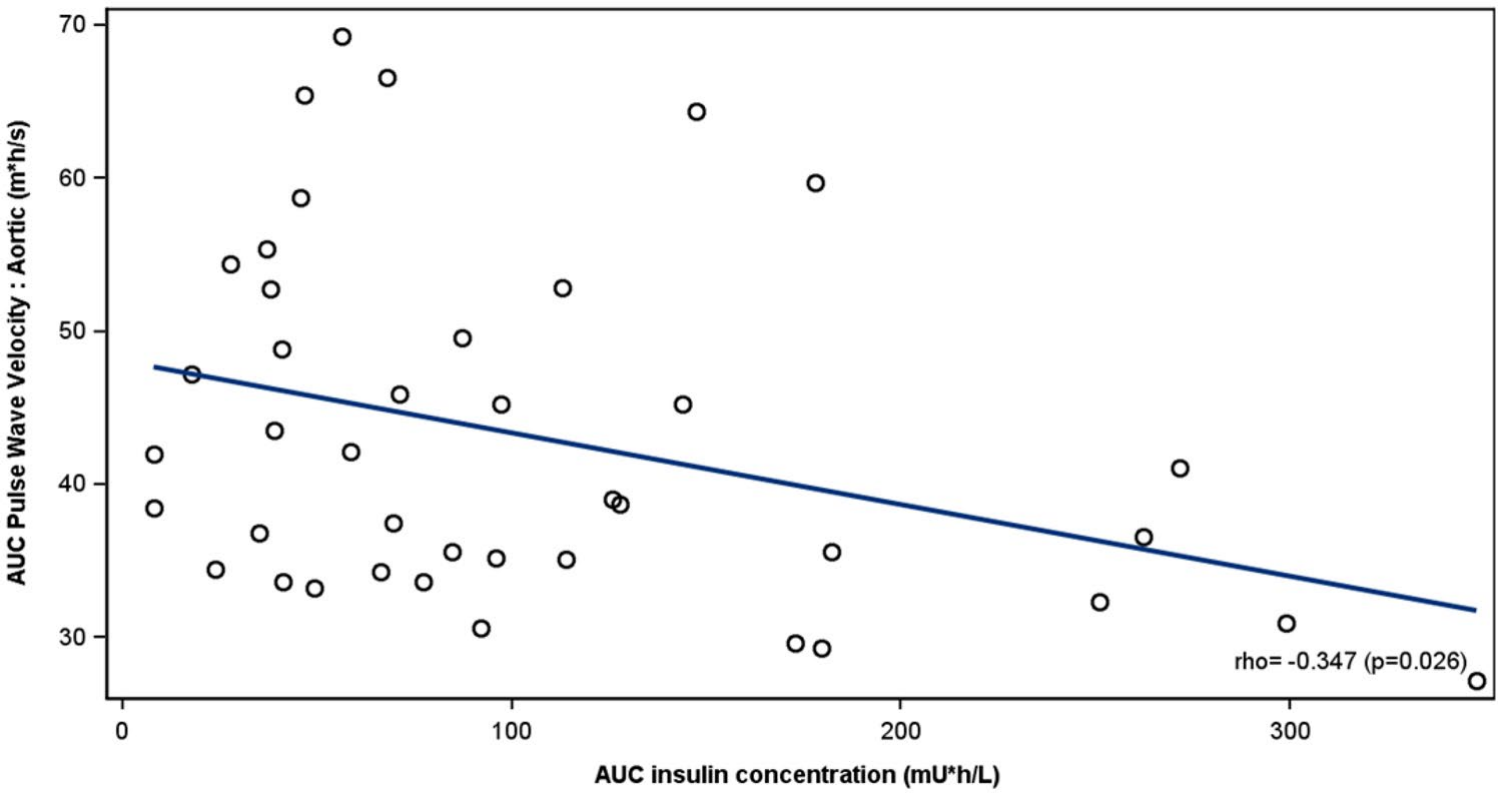
Correlation between AUC aortic PWV and AUC of endogenous serum insulin concentration in patients with type 2 diabetes with albuminuria. For each patient, a study with and without pre-meal lispro is included

**Fig. 4:**
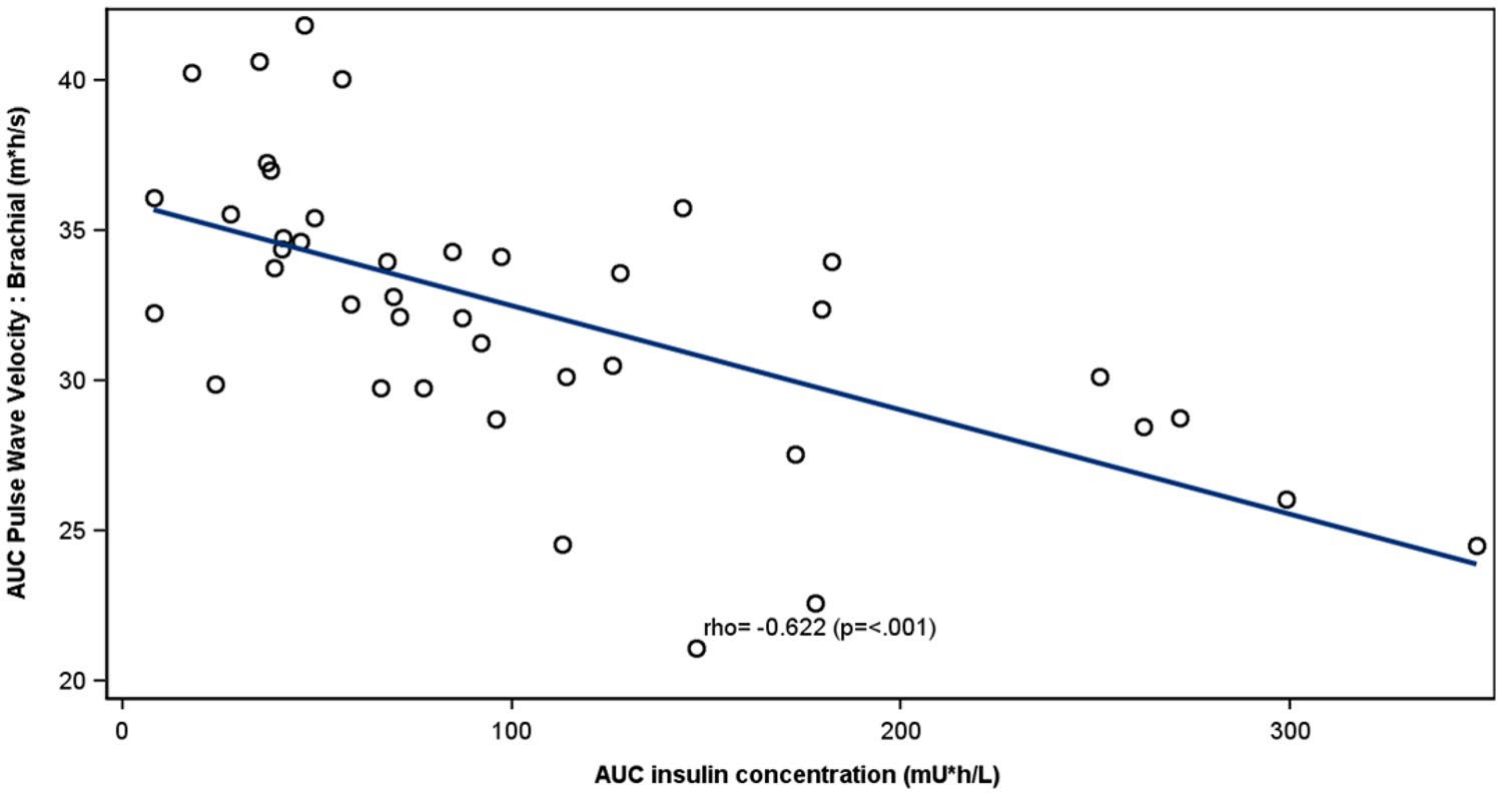
Correlation between AUC brachial PWV and AUC of endogenous serum insulin concentration in patients with type 2 diabetes with albuminuria. For each patient, a study with and without pre-meal lispro is included

In patients with albuminuria, there was a significant inverse correlation between total insulin dose/weight and AUC aortic (Fig. 1) and brachial PWV (Fig. 2). Similarly, there was an inverse relationship between AUC of endogenous serum insulin concentration and AUC aortic PWV (Fig. 3) and brachial PWV (Fig. 4). When glucose was included in these correlations (insulin dose/weight/AUC glucose vs AUC PWV), the significance remained in the albuminuric patients for aortic (*r *= − 0.383, *p *< 0.011) and brachial PWV (*r *= − 0.372, *p *< 0.014). Equally, the correlations after adjustment for serum glucose between the AUC serum insulin concentration/weight/AUC glucose and the AUC PWV were significant in the albuminuric patients for aortic (*r *= − 0.381, *p *< 0.014) and brachial PWV (*r *= − 0.571, *p *< 0.001). Inclusion of total cholesterol and eGFR in the model on top of the previous covariates did not influence the associations between insulin and arterial stiffness further strengthening our results (data not shown). Notably, in patients without albuminuria or in healthy controls no such correlations between insulin and PWV were observed. However, the slopes were significantly different between patients with and without albuminuria only in case of brachial PWV, as the interaction term between albuminuria groups and insulin was significant (*p *= 0.002 for both AUC insulin concentration, and total insulin dose). No interactions were observed between controls and either of the diabetes groups. The data were further separately analyzed for high- or low-postprandial glucose conditions. The associations between brachial PWV and insulin dose/weight during high- and low-postprandial conditions were *r *= − 0.543, *p* = 0.013 and − 0.659, *p *= 0.001, respectively. The associations between aortic PWV and insulin dose/weight during high -and low-postprandial conditions were 0.574, *p *= 0.008 and − 0.442, *p* = 0.039, respectively.

## Discussion

We observed an inverse correlation between insulin and PWV in the T2D patients with but not without albuminuria or in healthy controls. Microalbuminuria is associated with autonomic neuropathy [6, 7] and endothelial dysfunction [8]. Since insulin plays a key role in the regulation of arterial blood flow, tone and endothelial function [13], we examined whether albuminuria influences the association between insulin availability and postprandial PWV in large arteries.

The correlation between postprandial arterial stiffness (PWV) and insulin under hyperglycemic conditions in patients with T2D remained statistically significant, when insulin dose/weight or serum insulin concentration was adjusted for the plasma glucose concentration suggesting that the association is independent of the glycemia and may reflect a direct insulin-medicated effect. We had a specific assay for endogenous insulin, but did not have a specific assay for lispro or insulin glargine or detemir. For this reason, we used the insulin dose as a second measure of insulin availability. There was an inverse correlation between PWV and with both endogenous insulin as well as insulin dose, supporting our hypothesis of a correlation between insulin and PWV. Surprisingly, these correlations were neither present in T2D patients without albuminuria nor in healthy individuals. The significant difference between the albuminuric and the non-albuminuric patients regarding the correlation coefficients trends of insulin vs PWV further supports that insulin has a different effect on PWV in these two patient groups. A potential role of insulin is further supported by the fact that there was no difference between the groups regarding the prevalence of cardiovascular disease, cholesterol-lowering or antihypertensive medications. In this particular study, the original aim was to examine the effect of postprandial hyperglycemia on PWV. For this reason, we selected patients with type 2 diabetes, who were using basal–bolus regimens so that one study can be done with and another without pre-meal short-acting insulin. This design served well to examine a potential relationship of PWV and postprandial insulin, endogenous or exogenous. We could not observe differences in established biomarkers reflecting inflammation, endothelial function or advanced glycated end-products (sCRP, sICAM-1, sVCAM-1, ADMA, sRAGE) between the groups [9].

The finding may be explained by insulin’s dual role in the vasculature. Although insulin is a vasodilator, concomitantly it can also constrict the arteries through the autonomic nervous system [5]. The balance and the shift in the balance of the sympatico-excitatory and the vasodilator effects of insulin can occur already at modestly elevated plasma insulin levels [9], as our patients had. One could anticipate that patients with T2D and albuminuria have advanced autonomic neuropathy [6, 7], which can diminish the vasoconstrictive effects of insulin. As a result, the vasodilatory effects of insulin were dominant in the current setting resulting in a reduced PWV. Importantly, we can only speculate on these mechanisms as we lack direct measures of autonomic neuropathy in this study. Another contributing factor could be among patients with albuminuria a higher sensitivity to insulin and its vasodilatory effects as compared to patients without albuminuria. While patients were using exogenous insulin, their serum endogenous insulin concentrations were similar to healthy individuals, probably reflecting insulin resistance.

This study does not go without limitations. The study groups were relatively small and the correlations might be influenced by a few extreme observations. We have, therefore, presented the data in scatter plots showing the response of individual subjects. Regarding the insulin exposure, there were no differences in insulin doses or areas under the curves for endogenous insulin between albuminuric and non-albuminuric patients. Thus, differences in insulin exposure cannot explain the difference in the relationship of PWV and insulin in the two patient groups. We used the insulin dose as an approximate of total exogenous insulin exposure which may not be an exact measure. This approach was, however, consistently used throughout the study. We cannot exclude the fact that albuminuria would have been masked by the use of RAAS inhibitors in this real-life study. We did not have any specific measures of autonomic neuropathy, but it is well established that patients with albuminuria have a more advanced autonomic neuropathy than patients without albuminuria [6].

In conclusion, we observed an inverse correlation between insulin and arterial stiffness in large (aortic) and middle-sized (brachial) arteries measured as PWV in individuals with T2D and albuminuria. This was not observed in patients with T2D but without albuminuria or in non-diabetic individuals. Potentially, insulin counteracts the vasoconstriction caused by endothelial dysfunction and increased sympathetic activity known to be present in albuminuric patients with T2D. Interestingly, we observed a positive correlation between PWV and postprandial serum glucose in the same patients, and only again in the patients with albuminuria [9]. Thus, it is possible that in albuminuric patients, the arteries are sensitive to acute stiffening by glucose, and to relaxation by insulin.
